# Understanding resource use and dietary niche partitioning in a high-altitude predator guild using seasonal sampling and DNA metabarcoding

**DOI:** 10.1371/journal.pone.0315995

**Published:** 2024-12-19

**Authors:** Charlotte E. Hacker, Wei Cong, Yunchuan Dai, Jia Li, Ye Li, Diqiang Li, Rodney Jackson, Jan E. Janecka, Yuguang Zhang

**Affiliations:** 1 Ecology and Nature Conservation Institute, Chinese Academy of Forestry, Key Laboratory of Biodiversity Conservation of National Forestry and Grassland Administration Beijing, Beijing, China; 2 Department of Biological Sciences, Duquesne University, Pittsburgh, Pennsylvania, United States of America; 3 Snow Leopard Conservancy, Sonoma, California, United States of America; 4 Institute for Ecology and Environmental Resources, Chongqing Academy of Social Sciences, Chongqing, China; MARE – Marine and Environmental Sciences Centre, PORTUGAL

## Abstract

Understanding of predator feeding ecology, interactions among co-occurring predator species, and seasonal changes is critical for conservation management given the important role that predators play in shaping their ecosystems, but is lacking for most regions of the world. Dietary studies have demonstrated varying conclusions in the role that resource partitioning plays in the maintenance of predator communities due to complex inter-related factors that may shape prey use. We used DNA metabarcoding on 581 scat samples to determine the dietary composition, similarity, diversity, and niche overlap of eight predator species (Tibetan wolf (*Canis lupus*), snow leopard (*Panthera uncia*), Tibetan brown bear (*Ursus arctos pruinosus*), Eurasian lynx (*Lynx lynx*), Tibetan fox (*Vulpes ferrilata*), red fox (*V*. *vulpes*), Pallas’s cat (*Otocolobus manul*), and beech marten (*Martes foina*)) across four sampling periods (September 2019, December 2019, March 2020, July 2020) in the Gouli Nature Reserve located in Dulan County, Qinghai Province, China. We identified 26 unique prey items, with blue sheep (*Pseudois nayaur*) and pika (*Ochotona* spp.) being most common. Small mammals had the highest frequency of occurrence, while domestic and wild ungulates contributed the most biomass. No significant differences in diet were detected across months, with the exception of March and December for the red fox (*p* = 0.010). Dietary niche overlap was greater than expected when considering all species (*p* < 0.001) across seasons and between the Tibetan wolf and snow leopard in March (*p* = 0.007) when compared for species pairs by season. This study contributes to understanding of fine-scale temporal changes in predator diet, and offers methodological and management strategies that may have applicability to other predator guilds living in complex landscapes.

## Introduction

The understanding of carnivore diet composition, and the variables that influence it, is of high interest due to the role carnivores play in their respective ecosystems [1, 2]. Knowledge of diet can elucidate prey presence, key prey species [3], predation pressure [4], degree of livestock loss [5], niche differentiation [6], and intra- and interspecific competition [7].

Dietary niche partitioning studies have presented differing conclusions in the role that partitioning plays in the maintenance of carnivore communities. Differences in prey use was deemed responsible for the stable co-existence of wolves (*Canis lupus*) and coyotes (*Canis latrans*) in the United States [8]. Alternatively, similarities were observed between leopards (*Panthera pardus*), spotted hyena (*Crocuta crocuta*), brown hyena (*Parahyaena brunnea*), and wild dog (*Lycaon pictus*) in Botswana [9]. Overlap may be driven by a high abundance of prey that reduces competition between predator species [9]. In addition, smaller predators may take advantage of scavenging opportunities provided by kills made by larger predators [10].

Species in high-altitude environments, such as those on the Qinghai-Tibetan Plateau (QTP) of China, will have to withstand potential reduction in habitat, increased fragmentation, loss of dispersal corridors, and a higher frequency of conflicts with humans due to climate change and human activities [11]. The QTP is a 2.5 million km^2^ landscape which affects global weather patterns [12] and holds the headwaters for the Three Rivers, on which 40% of the world’s population depends on or is influenced by [13]. It has previously faced unprecedented environmental pressure [14], but policies and national parks are working to protect landscapes and encourage wildlife repopulation [14–17]. However, climate change remains a threat that will continue to cause reductions and distribution shifts of predators, their prey, and biodiversity, ultimately influencing animal behavior and ecosystem function. For example, changes in the dynamics or number of one predator species may impact others or predators may overlap with species they did not previously compete with, potentially reducing their effectiveness at procuring resources.

Apex predators living on the QTP include the Tibetan wolf, Tibetan brown bear (*Ursus arctos pruinosus*), snow leopard (*P*. *uncia*), and Eurasian lynx (*Lynx lynx*) [18]. Mesocarnivores include the red fox (*Vulpes vulpes*), Tibetan fox (*V*. *ferrilata*), Pallas’s cat (*Otocolobus manul*), Eurasian badger (*Meles meles*), and beech marten (*Martes foina*), among others. The most common native prey species include blue sheep (*Pseudois nayaur*), Tibetan gazelle (*Procapra picticaudata*), argali (*Ovis ammon*), white-lipped deer (Thorold’s deer; *Cervus albirostris*), Himalayan marmot (*Marmota himalayana*), chukar partridge (*Alectoris chukar*), and pika (*Ochotona* spp.) [19]. Livestock such as domestic yak (*Bos grunniens*), goat (*Capra aegagrus hircus*), sheep (*Ovis aries*), horse (*Equus caballus*), domestic pig (*Sus* scrofa), and camel (*Camelus ferus*) are common. Pastoralism remains an important source of livelihood, form of secondary income, or subsistence [20, 21]. Livestock can play a role in sustaining predators but livestock predation can cause conflict and great expense for local herders [22]. Loss of livestock can cause financial hardship, negative attitudes toward predator species, tension between pastoralists and entities seeking to protect wildlife, and in extreme cases, prompt retaliatory killings [23–25].

Previous work studying prey use of predators on the QTP detailed their diets via prey frequency of occurrence (FOO) and assessed dietary diversity, similarity, and niche overlap. Blue sheep (*Pseudois nayaur*) and pika (*Ochotona* spp.) were heavily represented in the data set, and livestock was found in five of the seven predator species studied [26]. Dietary overlap was statistically higher than expected based on null models, with only minor evidence of partitioning between apex predators and mesocarnivores. Interestingly, dietary breadth did not differ between apex predators and mesocarnivores, with the exception of the red fox. Thus challenging the commonly held notion that apex predators are typically more specialized.

What is grossly missing from studies in this area is the impact of season on prey use and dietary niche overlap. Better understanding of seasonal impacts can help close many of knowledge gaps surrounding the functioning of this predator guild. For example, mesocarnivores may take advantage of smaller newborn calves available during birthing season in addition to their standard dietary repertoire, thereby increasing mesocarnivore dietary breadth. Alternatively, mesocarnivores may limit consumption of their typical prey as they take advantage of newborn calves, thereby decreasing mesocarnivore dietary breadth [27]. Lower ecologically productive environments in winter months may encourage predators to pursue livestock when wild prey are less abundant or available. Data surrounding livestock predation can assist in designing the non-lethal management tools necessary to reduce future loss [28]. In addition, the QTP is an area sensitive to climate change. The mountainous interior of Asia is warming at twice the rate of nonactic regions in the Northern Hemisphere [29]. Permafrost on the QTP spans the largest continuous area in the world, and nearly one-fifth of that total was lost between 1960 and 2009 [30]. Climate change will likely initiate large-scale events on the QTP that cause reductions and distribution shifts of predators and their prey. For example, the survival and timing of hibernating species may change, breeding seasons for small mammals may extend, or predator species who did not previously overlap will find themselves competing for resources [31].

Detail surrounding biomass consumed is also lacking in previous studies of predators on the QTP. Prey FOO in predator diets can be ecologically uninformative and skew data interpretation. For example, FOO weighs small and large prey items identified in scat samples equally and can overestimate the role that small prey items play [32]. Linear regression models using feeding trials have been developed for numerous predator species and can be used to calculate biomass [33]. One such example is a linear regression model designed for the wolf (Y = 0.439 + 0.008X), where X is the mean mass of a prey item and Y is the mass of the prey item per scat. Despite its shortcomings, FOO remains a valuable metric for comparison to previously collected FOO data, as well as to document unexpected prey items [26]. It is possible that the percentages of dietary contribution for each species using FOO data and biomass data may not be statistically significant different from one another, in which case the same conclusions would be drawn to inform conservation decision making regardless.

Most prey use studies have relied on traditional approaches such as microhistology [8, 34–38]. Traditional dietary analyses face several challenges. They are time consuming, subject to bias, may misidentify the predator that deposited the scat, and are dependent on the presence of physical prey matter (e.g., hair, teeth, bones) in the sample [39, 40]. Next-Generation Sequencing can overcome these limitations. With DNA metabarcoding, the organisms in each scat are identified by PCR amplification and sequencing of a diagnostic gene segment with its origin determined by bioinformatically matching its sequence to a reference database [39]. Mitochondrially encoded 12S rRNA (MT-RNR1) serves as a genetic marker for this purpose and has previously shown utility for species found on the QTP [26, 41].

To elucidate how predators on the QTP overlap in diet and how seasonality influences this, we non-invasively sampled 10 fixed transects across four seasons for one year in the Gouli Nature Reserve (GNR), a protected area in Dulan County, Qinghai Province, China. The goals of this study were to (1) determine the dietary composition (both wild and domestic prey) of predator species using DNA metabarcoding and calculate the frequency of occurrence (FOO) of species observed in diet as well as biomass contribution of prey species observed, (2) assess how dietary FOO and biomass seasonally changes by comparing these values across four different periods, and (3) quantify dietary similarity and niche overlap using Jaccard’s similarity index and Pianka’s index. Our results will help discern the key prey that may warrant focused conservation to ensure persistence, the importance of dietary niche partitioning for this predator guild, reliance on livestock, and how seasonal knowledge of diet can be leveraged to tailor conservation and management decisions with regard to temporal variation.

## Materials and methods

This study was carried out on protected land and all species were noninvasively sampled. All necessary permits and permissions were obtained from the Qinghai Wildlife Management and Protection Bureau and the Dulan County Forestry Reserve before the study began.

### Study site

Gouli Township, located on East Burhanbuda Mountain of the Kunlun Mountains in Dulan County, Qinghai Province, China, measures 2,559.4 km^2^. It holds the Gouli Nature Reserve (GNR) (35.659528°N, 98.499298°E) and has human inhabitants. Topography consists mostly of grassland with rock slopes [42]. Residents are predominately Tibetan semi-nomadic pastoralists keeping livestock in summer pastures at approximately 4,600 m in elevation with winter and spring pastures at altitudes of around 4,000 m [24]. The climate is characterized by long, dry, cold winters with strong winds and solar radiation [12, 43]. During this study, the mean temperature ranged from -8.17°C in December 2019 to 15.78°C in July 2020. Precipitation was highest in June 2020 with 73 mm and lowest in January 2020 with 1.78 mm (NOAA, https://www.ncdc.noaa.gov/). A total of 137 scat samples were collected along 20 km of transects in July 2018 for a separate study [26]. Results using similar methodology and analyses quantitatively confirmed a diverse predator guild of 7 different species and qualitatively supported the presence of an abundant prey base present in the GNR predominately comprised of species such as blue sheep (*Pseudois nayaur*), Himalayan marmot (*Marmota himalayana*), pika (*Ochotona* spp.), domestic yak (*Bos grunniens*), and sheep (*Ovies aries*), and this was selected as a suitable site for the present study.

### Transect selection

**Fig 1:**
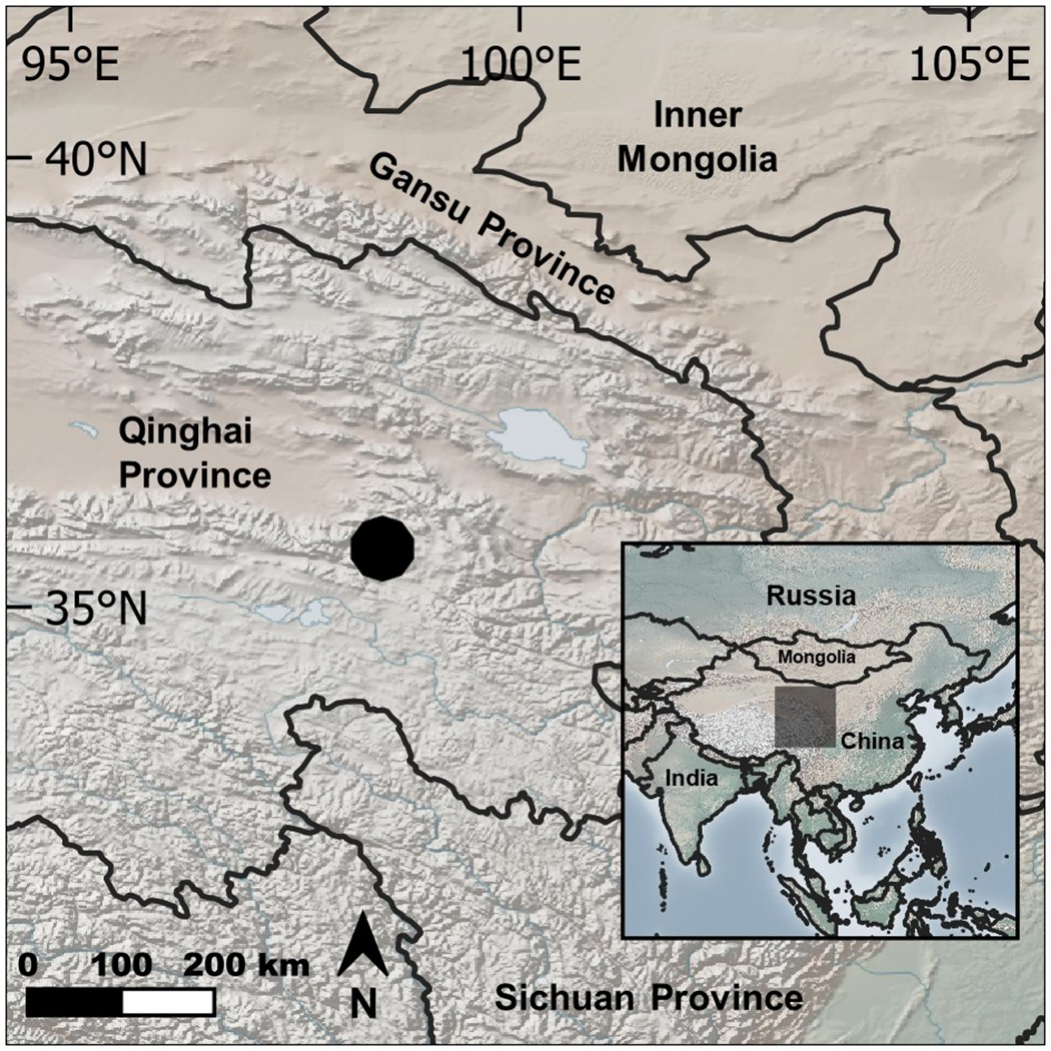
The location of the ten fixed transects sampled for scat in the Gouli Nature Reserve, Dulan County, Qinghai Province, China in September 2019, December 2019, March 2020, and July 2020. Map raster data obtained from Natural Earth (https://www.naturalearthdata.com/).

Transects for the present study were based on predator presence confirmed by camera trap or observations by local herders within the previous 4 months. Additional factors considered were year-round accessibility (i.e., not prone to flooding, winter access), seasonal grazing status (i.e., seasons with livestock present), and relative location among four areas within Gouli Township–Ren Long Cun of Gouli, Duo Jiao Hu, Delong Guo, and Re Long Guo (i.e., to avoid one transect being adjacent to another). This resulted in ten transects totaling 22.5 km in length (Fig 1, S1 Table).

### Sample collection

Sample collection followed previous published materials and protocols [44, 45]. Only samples that appeared to be “fresh” or “near-fresh” were collected [44]. Using new gloves for each scat sample, a full-cross-section from the freshest appearing portion of the scat was broken off and placed into a labeled 15 mL tube with 7 mL of color indicated silica desiccant. Approximately ½ to ¾ of the volume of the whole sample was left in the field so as to not disrupt chemical communication and marked with colored nail polish to help determine degradation time from observations at later dates. Samples were stored at -20°C until DNA extraction. A total of 188 scat samples were collected in September 2019, 130 in December 2019, 133 in March 2020, and 130 in July 2020, resulting in 581 scat samples for all four periods.

### DNA extraction

DNA was extracted using QIAamp DNA Stool Mini Kits [QIAGEN, Hilden, Germany] following standard protocols with two additional centrifugation steps to remove residual Buffer AW1 and AW2. Aliquots were quantified using a NanoDrop Lite Spectrophotometer.

### PCR for species and diet analysis

Host (i.e., consumer) species were identified via amplification and sequencing of a ~100-bp fragment of the V5 loop of the MT-RNR1 gene using a universal primer for vertebrates 12SV5F/12SV5R [41]. The segment was amplified in a PCR reaction containing 1.5 μL of DNA template, 7.94 μL of KAPA HiFi HotStart ReadyMix (2X) [Kapa Biosystems, Wilmington, MA, USA], 0.16 μL of 20 μM forward primer, 0.16 μL of 20 μM reverse primer, and 5.2μL of PCR grade water. PCR conditions were 95 ºC for 3 min, 35 cycles of 95 ºC for 30 sec, 60 ºC for 30 sec, and 72 ºC for 30 sec, followed by 5 min extension step at 72 ºC and 4 ºC hold.

### Sequencing

Amplicons were mixed in equidensity ratios determined by measuring gel band brightness using GeneTools Analysis Software Version 4.03.05.0 [SynGene, Frederick, MD, USA]. The E.Z.N.A.^®^ Gel Extraction Kit [Omega Bio-Tek, Inc., Norcross, GA, USA] was used to extract and purify MT-RNR1 products. The NEBNext^®^ Ultra^™^ II DNA Library Prep Kit for Illumina^®^ [New England Biolabs, MA, USA] was used to prepare sequencing libraries following manufacturer’s recommendations. Index codes were added to incorporate unique barcodes to amplicons from different samples. The ampliconic library was quantified using an Invitrogen Qubit^®^ 2.0 Fluorometer [Thermo Fisher Scientific, Waltham, MA, USA]. Paired end 250-bp sequencing was completed on an Illumina NovaSeq 6000 by Guangdong Magigene Biotechnology Co., Ltd. [Guangzhou, China].

### Diet analysis

Potential prey items were identified via a literature search of prey common to predator species residing on the QTP and consultation with local experts [35, 46–48]. MT-RNR1 reference sequences for potential prey items were pulled from NCBI GenBank (https://www.ncbi.nlm.nih.gov/) or the Barcode of Life Data System (Bold System v4, http://www.barcodinglife.org/). FASTQ reads were extracted and put into a concatenated file. They were demultiplexed, adapters removed, and imported into CLC Genomics Workbench v12.0 [CLC bio, QIAGEN, Aarhus, Denmark]. Raw reads were trimmed using a quality score limit of 0.1 and mapped to the reference FASTA file using local alignment with the following parameters—mismatch cost: 2; insertion cost: 3; deletion cost: 3; length fraction: 0.9; similarity: 0.94; non-specific matches mapped randomly. Samples were considered “Undetermined” if the alignments in CLC Genomics Workbench v12.0 showing mapped reads to host predator species reference sequences could not be interpreted due to lack of data and low similarity, suggesting that there were an insufficient number of matching reads. Prey identification was made based on reference taxa that had the highest number of mapped reads at >98% similarity with the fewest mismatches. Samples with sequencing reads mapping to more than two potential host species were deemed “Inconclusive,” but were first examined for possible cases of intraguild predation considering the following: sequencing reads mapped to only two potential host species with no reads mapped to a potential prey item, and unmapped reads constituted <5% of total reads to ensure that another unknown species absent from the reference file was not a possible missed non-carnivore species prey item. The host species with the higher number of reads was considered the species that consumed the prey [26]. To ensure prey was correctly identified, its consensus sequence, constructed using the most common nucleotides at each position among all mapped reads to that species was extracted and blasted against nucleotide databases with *blastn*. Species that had greatest similarity to the consensus sequence were compared to the result from the mapping-to-reference method. The 100-bp fragment of the V5 loop of MT-RNR1 (12S) is known to have ambiguity among caprids found on the QTP, particularly domestic sheep and argali (*O*. *ammon*) [49]. The GNR is positioned on the border of where argali populations are considered “extinct” and “possibly extant (resident)” according to the geographic range map published for the species under their IUCN Red List assessment [50]. Local experts confirmed that argali were not in the study area, and thus sequence ambiguity between the species was not anticipated to be an issue.

Samples in which prey could not be discerned had their unmapped reads analyzed to rule out an incomplete reference file. This was done by performing a *de novo* assembly with the following parameters—minimum contig length of 100; mismatch cost: 2; insertion cost: 3; deletion cost: 3; length fraction: 0.9; similarity: 0.98. Consensus sequences were extracted for contigs with the highest number of mapped reads. Low coverage definition threshold was set at 10,000. Nucleotides in sites with conflicting reads were resolved via majority rule and ambiguous sites coded with an “N.” Samples that did not map consensus sequences to any prey species were categorized as “Unknown.”

### Data analysis

All statistical analyses were performed in Microsoft Office Excel for Mac Version 16.43 and R version 3.5.2 (R Core Team 2018) using base R functions, as well as the *iNEXT* [51] and *EcoSimR* packages [52]. Host species composition percentages were calculated by summing the number of scats belonging to each host (s) and dividing it by the total number of scats (S) (s/S*100).

In order to determine the dietary composition of host predators based on prey frequency of occurrence (FOO), the number of occurrences of a unique prey species (r) was divided by the total number of prey items (R; i.e., all scat assigned to that predator) and converted into a percentage (r/R*100). Sample completeness with respect to sample size for host predator species was calculated with a process of rarefaction using the ‘iNEXT’ function. The number of scat samples with more than one prey item were summed and assigned to host. Prey species were grouped hierarchically into a Primary Tier (species name), Secondary Tier (group name), and Tertiary Tier (category name) (e.g., brown accentor (*Prunella fulvescens*), perching birds, birds) for data interpretation purposes (S2 Table).

In order to determine the dietary composition of host predators based on the biomass contribution of each prey item, a biomass calculation model was used. Biomass calculation models derived from feeding trials appear to be the best approximation of true predator diet. Prey biomasses were determined from peer-reviewed literature (S4 Table). The biomass consumed by each host was calculated using a linear regression model designed for the wolf based on feeding trials [53] with modifications (Y = 0.439 + 0.008X) [32] as in similar diet studies [54]. Where X is the mean mass of a prey item and Y is the mass of the prey item per scat. Y was multiplied by the number of occurrences of each prey item to estimate its contribution to diet. The percentages calculated for prey FOO and biomass were compared using a Wilcoxon signed rank test to determine the comparative similarity between the two.

Three different metrics, richness, the Shannon-Wiener index, and the Simpson’s index, were used to examine host predator dietary diversity overall and by season. Richness was calculated by summing the number of unique prey species. The total number of prey species identified and the proportion of each species present in the host predator’s diet were used to calculate the Shannon-Wiener index [55] and Simpson’s index [56]. A higher resulting value for the Shannon-Wiener index indicates higher dietary diversity while a lower value for the Simpson’s index indicates higher dietary diversity.

Seasonal differences in prey FOO and biomass were calculated with a Friedman’s test using the four seasons as independent repeated measures for species, and species as independent repeated measures for season. A Wilcoxon signed rank test was used to identify significant differences between any two months in a single predator’s diet for each prey species when sample sizes allowed.

The Jaccard’s similarity index was used to quantitatively assess dietary similarity across the predator guild, between any two given host predator species, and between any two given seasons in one host predator based on prey presence or absence in scat. Calculated values were subtracted from 1 to obtain Jaccard’s similarity values with higher values being indicative of greater dietary similarity between two species. Jaccard’s similarity values were visualized using multidimensional scaling via principal coordinates analysis.

Pianka’s index was used to calculate dietary niche overlap using FOO data across the predator guild and between any two given host predator species [57, 58]. Pianka’s index values range from 0 to 1, with higher values being indicative of more dietary overlap. This value was compared to 999 null model simulations using the ‘niche_null_model’ function in the *EcoSimR* package to determine if dietary niche overlap was more or less than expected if each predator species used prey items independently of one another [52].

## Results

### Host and prey species identification

**Fig 2:**
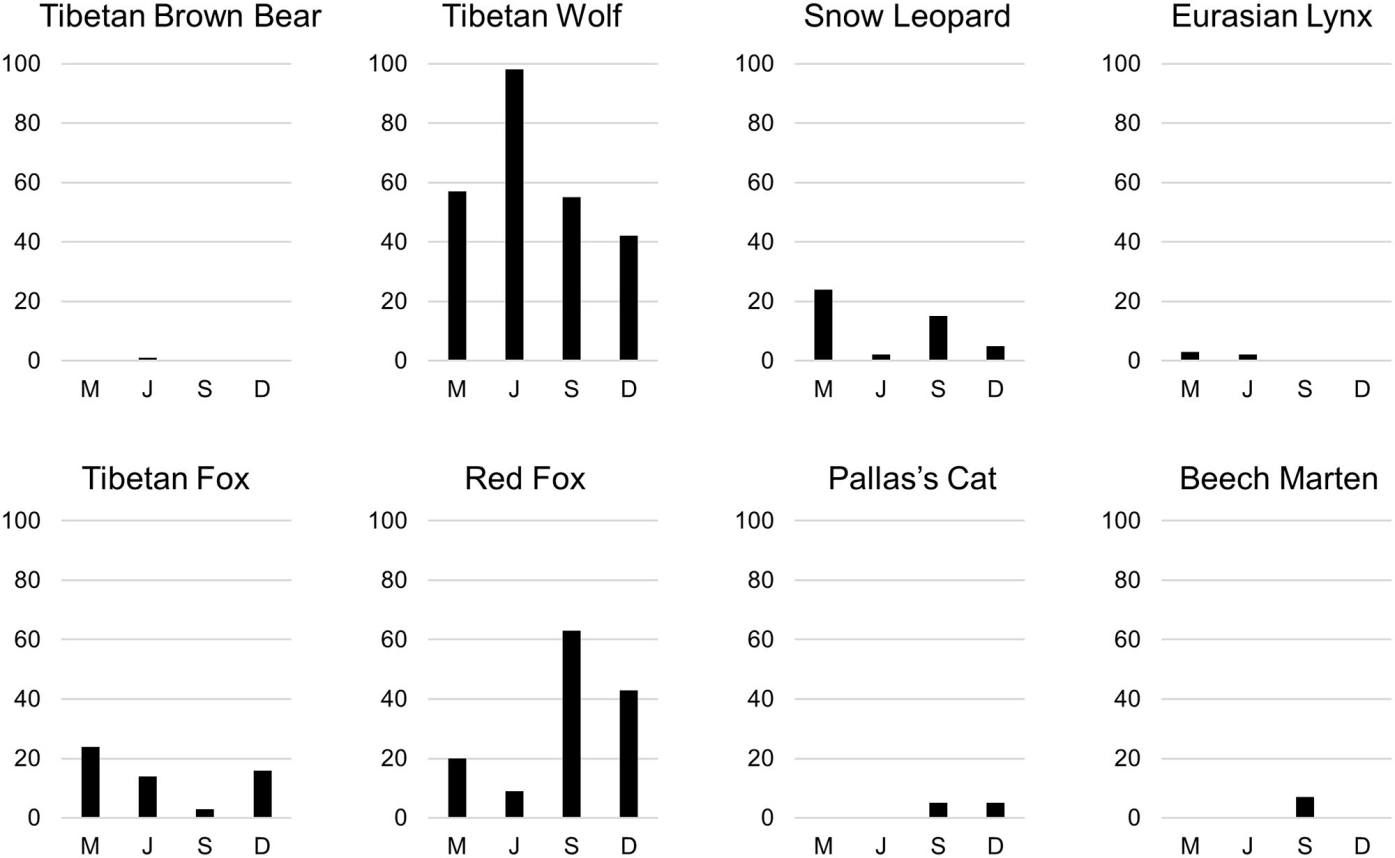
The actual number of scats genetically determined to belong to each identified host predator species by month (M = March, J = July, S = September, D = December) in the Gouli Nature Reserve, Dulan County, Qinghai Province, China in September 2019, December 2019, March 2020, and July 2020.

Out of 581 scat samples, 7 were regurgitated bird pellets (upland buzzard *Buteo hemilasius*), Eurasian eagle owl *Bubo bubo*, falcon *Falco* spp.), 1 was from a non-target species (white-lipped deer *Cervus albirostris*), 8 were Inconclusive, and 54 were Undetermined. This left 511 samples from 8 terrestrial predator species (Table 1, Fig 2, S3 Table). Most belonged to Tibetan wolf (252 samples), followed by red fox (135 samples), then Tibetan fox (56 samples), snow leopard (46 samples), Pallas’s cat (10 samples), beech marten (6 samples), Eurasian lynx (5 samples), and Tibetan brown bear (1 sample).

**Table 1:** The sample sizes and percentages (%) of frequency of occurrence (FOO) for prey items in the diets of 8 predators across 4 seasons. (O = Overall, M = March, J = July, S = September, D = December). Scientific names and groupings are available in S2 Table. Note that Tibetan brown bear and beech marten were excluded due to low samples sizes and available dietary data for only one season.

Predator	Tibetan Wolf					Snow Leopard					Eurasian Lynx			Tibetan Fox					Red Fox					Pallas’s Cat		
No. of scats	252	57	98	55	42	46	24	2	15	5	5	3	2	56	23	14	3	16	135	20	9	63	43	10	5	5
Season	O	M	J	S	D	O	M	J	S	D	O	M	J	O	M	J	S	D	O	M	J	S	D	O	S	D
Domestic Yak	11.0	15.9	14.1	0.0	5.7	4.3	7.7	0.0	0.0	0.0	0.0	0.0	0.0	2.9	3.4	5.6	0.0	0.0	4.2	8.0	5.9	3.0	3.3	0.0	0.0	0.0
Domestic Camel	0.3	0.0	0.0	2.0	0.0	0.0	0.0	0.0	0.0	0.0	0.0	0.0	0.0	0.0	0.0	0.0	0.0	0.0	0.0	0.0	0.0	0.0	0.0	0.0	0.0	0.0
Domestic Goat	3.2	3.7	4.0	0.0	3.8	0.0	0.0	0.0	0.0	0.0	0.0	0.0	0.0	0.0	0.0	0.0	0.0	0.0	0.6	0.0	0.0	0.0	1.7	0.0	0.0	0.0
Domestic Sheep	3.2	7.3	3.4	0.0	0.0	0.0	0.0	0.0	0.0	0.0	0.0	0.0	0.0	0.0	0.0	0.0	0.0	0.0	0.0	0.0	0.0	0.0	0.0	0.0	0.0	0.0
Blue Sheep	39.2	57.3	44.1	5.0	34.0	59.6	73.1	100.0	28.6	60.0	28.6	33.3	25.0	4.3	3.4	11.1	0.0	0.0	10.1	20.0	17.6	4.5	10.0	0.0	0.0	0.0
White-lipped Deer	0.3	0.0	0.0	1.7	0.0	0.0	0.0	0.0	0.0	0.0	0.0	0.0	0.0	0.0	0.0	0.0	0.0	0.0	0.0	0.0	0.0	0.0	0.0	0.0	0.0	0.0
Tibetan Fox	1.9	2.4	0.0	1.7	7.5	0.0	0.0	0.0	0.0	0.0	0.0	0.0	0.0	0.0	0.0	0.0	0.0	0.0	0.0	0.0	0.0	0.0	0.0	0.0	0.0	0.0
Red Fox	3.8	0.0	1.1	0.0	22.6	2.1	0.0	0.0	0.0	20.0	0.0	0.0	0.0	0.0	0.0	0.0	0.0	0.0	0.0	0.0	0.0	0.0	0.0	0.0	0.0	0.0
Hima-layan Marmot	13.7	0.0	15.3	33.3	7.5	4.3	0.0	0.0	14.3	0.0	14.3	0.0	25.0	4.3	3.4	5.6	0.0	5.6	4.8	0.0	17.6	7.6	0.0	0.0	0.0	0.0
Woolly Hare	1.6	0.0	1.7	1.7	3.8	0.0	0.0	0.0	0.0	0.0	14.3	33.3	0.0	0.0	0.0	0.0	0.0	0.0	3.0	0.0	5.9	1.5	5.0	0.0	0.0	0.0
Zokor	0.3	0.0	0.0	0.0	1.9	2.1	0.0	0.0	7.1	0.0	0.0	0.0	0.0	1.4	0.0	0.0	0.0	5.6	0.6	0.0	0.0	1.5	0.0	0.0	0.0	0.0
Mountain Weasel	0.0	0.0	0.0	0.0	0.0	0.0	0.0	0.0	0.0	0.0	0.0	0.0	0.0	0.0	0.0	0.0	0.0	0.0	0.0	0.0	0.0	0.0	0.0	0.0	0.0	0.0
Pika	11.8	9.8	14.1	8.3	11.3	12.8	7.7	0.0	21.4	20.0	42.9	33.3	50.0	79.7	79.3	77.8	50.0	88.9	53.6	48.0	52.9	53.0	56.7	90.0	80.0	100.0
Long-tailed Dwarf Hamster	0.3	0.0	0.0	1.7	0.0	0.0	0.0	0.0	0.0	0.0	0.0	0.0	0.0	0.0	0.0	0.0	0.0	0.0	2.4	0.0	0.0	0.0	6.7	0.0	0.0	0.0
Vole Species	1.3	2.4	1.7	0.0	0.0	0.0	0.0	0.0	0.0	0.0	0.0	0.0	0.0	2.9	6.9	0.0	0.0	0.0	9.5	24.0	0.0	4.5	11.7	0.0	0.0	0.0
Common Shrew	0.0	0.0	0.0	0.0	0.0	0.0	0.0	0.0	0.0	0.0	0.0	0.0	0.0	0.0	0.0	0.0	0.0	0.0	0.6	0.0	0.0	1.5	0.0	0.0	0.0	0.0
Birds of Prey	0.0	0.0	0.0	0.0	0.0	0.0	0.0	0.0	0.0	0.0	0.0	0.0	0.0	1.4	3.4	0.0	0.0	0.0	0.6	0.0	0.0	1.5	0.0	0.0	0.0	0.0
Perching Birds	0.0	0.0	0.0	0.0	0.0	0.0	0.0	0.0	0.0	0.0	0.0	0.0	0.0	1.4	0.0	0.0	25.0	0.0	1.8	0.0	0.0	3.0	1.7	0.0	0.0	0.0
Ground Feeding Birds	1.1	1.2	0.0	3.3	1.9	0.0	0.0	0.0	0.0	0.0	0.0	0.0	0.0	0.0	0.0	0.0	0.0	0.0	4.2	0.0	0.0	7.6	3.3	0.0	0.0	0.0
Undeter-mined	7.3	0.0	0.6	41.0	0.0	14.9	11.5	0.0	28.6	0.0	0.0	0.0	0.0	1.4	0.0	0.0	25.0	0.0	4.2	0.0	0.0	10.6	0.0	10.0	20.0	0.0

**Fig 3:**
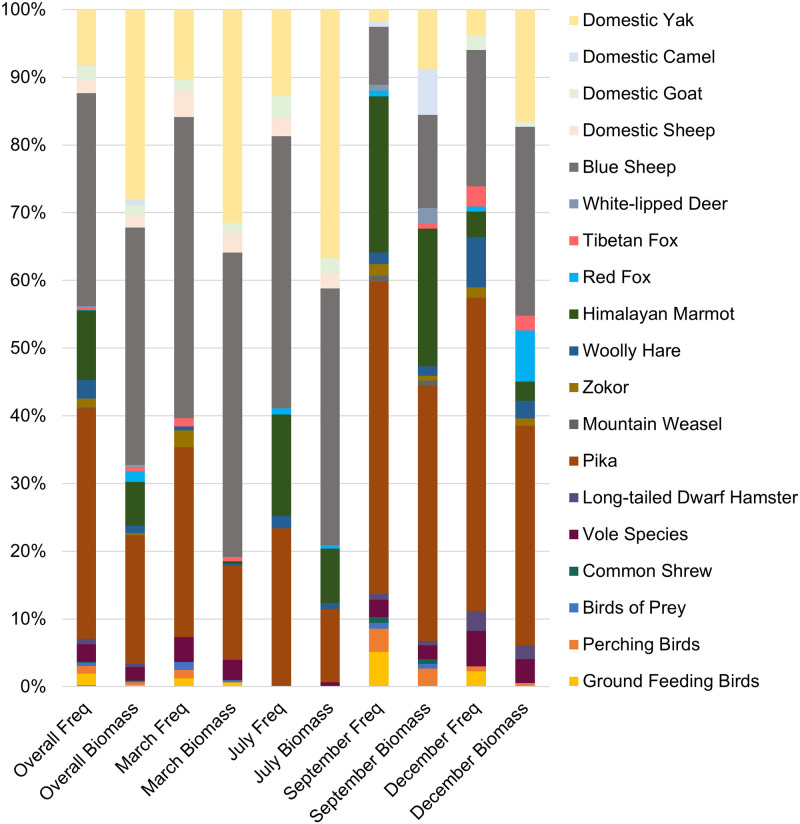
The relative percentages for dietary frequency (FOO) and biomass overall and by month for all 8 host predators in the study.

The number of unique host predators was consistent across months with sampling completeness showing overall satisfactory collection effort (S5 Table). There were 26 unique prey species from 2 animal classes (Mammalia, Aves) (S3 Table). Among all prey items identified in the diets of the 8 predator species in this predator guild, small mammals made up the largest frequency of occurrence (FOO) percentage, with pika dominating, while domestic and wild ungulates constituted the greatest biomass percentages (Table 1, Fig 3, S2 and S6 Tables). The 1 Tibetan brown bear scat contained a Himalayan marmot sampled in July (FOO– 100%, biomass– 100%). The Tibetan brown bear was removed from subsequent analyses given its isolated occurrence. The 6 beech marten scats were all sampled in September and contained 1 occurrence of mountain weasel (*Mustela altaica*) (FOO– 12.5%, biomass– 5.5%), 5 occurrences of pika (FOO– 62.5%, biomass– 19.2%), 1 occurrence of pine bunting (*Emberiza leucocephalos*) (FOO– 12.5%, biomass– 0.7%), and 1 occurrence of snowcock (*Tetraogallus himalayensis*) (FOO– 12.5%, biomass– 74.5%).

The Tibetan brown bear scat had 1 prey item. A mean of 1.42 prey species/scat (± 0.85) was found for Tibetan wolf, 0.98 (± 0.54) for snow leopard, 1.40 (± 0.89) for Eurasian lynx, 1.18 (± 0.43) for Tibetan fox, 1.51 (± 0.81) for red fox, 0.90 (± 0.32) for Pallas’s cat, and 1.57 (± 0.79) for beech marten. The maximum number of different prey species found in a single scat sample was 3.

### Dietary frequency versus biomass

There were no significant differences between FOO and biomass percentages overall (Z = -1.108, *p* = 0.268), for March (Z = -1.924, *p* = 0.054), July (Z = -1.305, *p* = 0.192), September (Z = -0.616, *p* = 0.538), or December (Z = -0.874, *p* = 0.382) (Fig 3, S6 Table).

### Dietary diversity

**Fig 4:**
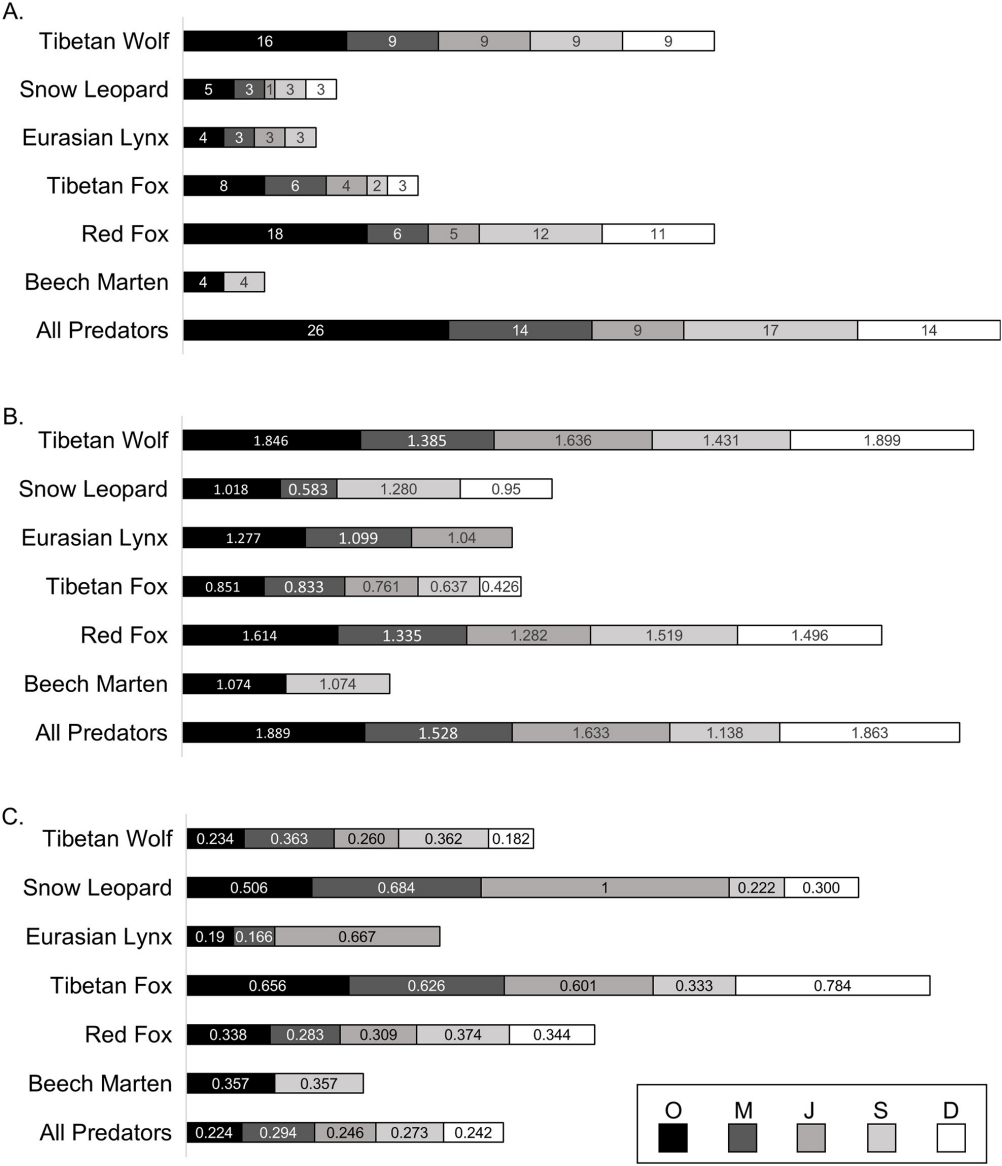
Horizontal bar plots representative dietary diversity for each host predator species and all host predators considered together where sample sizes allowed (O = Overall, M = March, J = July, S = September, D = December). (A) The number of unique dietary items (richness) identified in diet. (B) Dietary diversity as calculated by the Shannon-Wiener index, with a higher value equating to higher diversity. (C) Dietary diversity as calculated by the Simpson’s index, with a lower value equating to higher diversity.

Dietary richness ranged from 1 prey item for Pallas’s cat to 18 for red fox. Pallas’s cat was removed from calculations for the Shannon-Wiener index and Simpson’s index due to lack of prey diversity. Tibetan wolf had the highest diversity according to the Shannon-Wiener index and the Eurasian lynx for Simpson’s index (Fig 4).

### Seasonal changes in diet

When considering all host predators, diets across months were not significantly different for FOO (_*X*_^2^ (3) = 2.893, *p* = 0.408) or biomass (_*X*_^2^ (3) = 3.663, *p* = 0.300). Tibetan wolf had sufficient sample sizes to investigate diet across all months, and differences were found to be insignificant for FOO (_*X*_^2^ (3) = 3.643, *p* = 0.303) and biomass (_*X*_^2^ (3) = 2.023, *p* = 0.568). March, July, and December were compared for the Tibetan fox with no significant differences for FOO (_*X*_^2^ (2) = 2.800, *p* = 0.247) or biomass (_*X*_^2^ (2) = 0.080, *p* = 0.961). March and September were compared for snow leopard and were not significantly different for FOO (Z = -0.272, *p* = 0.785) or biomass (Z = 0.000, *p* = 1). March, September, and December were compared for the red fox, with FOO differences being statistically significant (_*X*_^2^ (2) = 7.048, *p* = 0.029) but not biomass (_*X*_^2^ (2) = 2.261, *p* = 0.323). Post-hoc tests revealed significant FOO differences between March and December (Z = 2.585, *p* = 0.010) but not March and September (Z = 1.490, *p* = 0.136), or September and December (Z = -0.159, *p* = 0.874).

### Dietary similarity and niche overlap

Beech marten, Eurasian lynx, and Pallas’s cat were removed from Jaccard coefficient analyses due to low sample sizes. Resulting Jaccard coefficients among the four remaining host predator species (Tibetan wolf, snow leopard, Tibetan fox, red fox) revealed varying values of dietary similarity between any two given species pairs overall or by season (Table 2, S7 Table).

**Table 2:** Jaccard’s similarity index for dietary similarity overall and across all seasons for all host predator species with appropriate sample sizes for comparisons. A higher value indicates greater similarity. (TW–Tibetan wolf; SL–snow leopard; TF–Tibetan fox; RF–red fox). Note that values denoted with ^✛^ represent those with low sample sizes in which resulting numbers should be interpreted with caution.

		Overall				March				July		September			December		
		TW	SL	TF	RF	TW	SL	TF	RF	TW	TF	TW	SL	RF	TW	TF	RF
Overall	TW	-															
	SL	0.400	-														
	TF	0.353	0.556	-													
	RF	0.556	0.357	0.615	-												
Mar.	TW	0.533	0.273	0.333	0.400	-											
	SL	0.200	0.500	0.375	0.231	0.375	-										
	TF	0.313	0.500	0.750	0.462	0.400	0.500	-									
	RF	0.333	0.375	0.444	0.385	0.625	0.600	0.571	-								
Jul.	TW	0.600	0.500^✛^	0.417	0.467	0.545	0.333	0.500	0.400	-							
	TF	0.267	0.667^✛^	0.500	0.308	0.333	0.750	0.667	0.500	0.444	-						
Sept.	TW	0.600	0.250	0.214^✛^	0.375	0.308	0.200	0.250	0.273	0.286	0.300	-					
	SL	0.267	0.667	0.500^✛^	0.308	0.200	0.400	0.429	0.286	0.300^✛^	0.600^✛^	0.300	-				
	RF	0.444	0.417	0.727^✛^	0.846	0.357	0.273	0.545	0.455	0.429	0.364	0.333	0.364	-			
Dec.	TW	0.667	0.600^✛^	0.385	0.533	0.500	0.300	0.333	0.364	0.583	0.400	0.462	0.400	0.500	-		
	TF	0.200	0.500^✛^	0.375	0.231	0.100	0.200	0.286	0.143	0.200	0.400	0.200	0.750	0.273	0.300	-	
	RF	0.500	0.250^✛^	0.417	0.692	0.545	0.333	0.364	0.556	0.500	0.300	0.385	0.182	0.538	0.462	0.091	-

**Fig 5:**
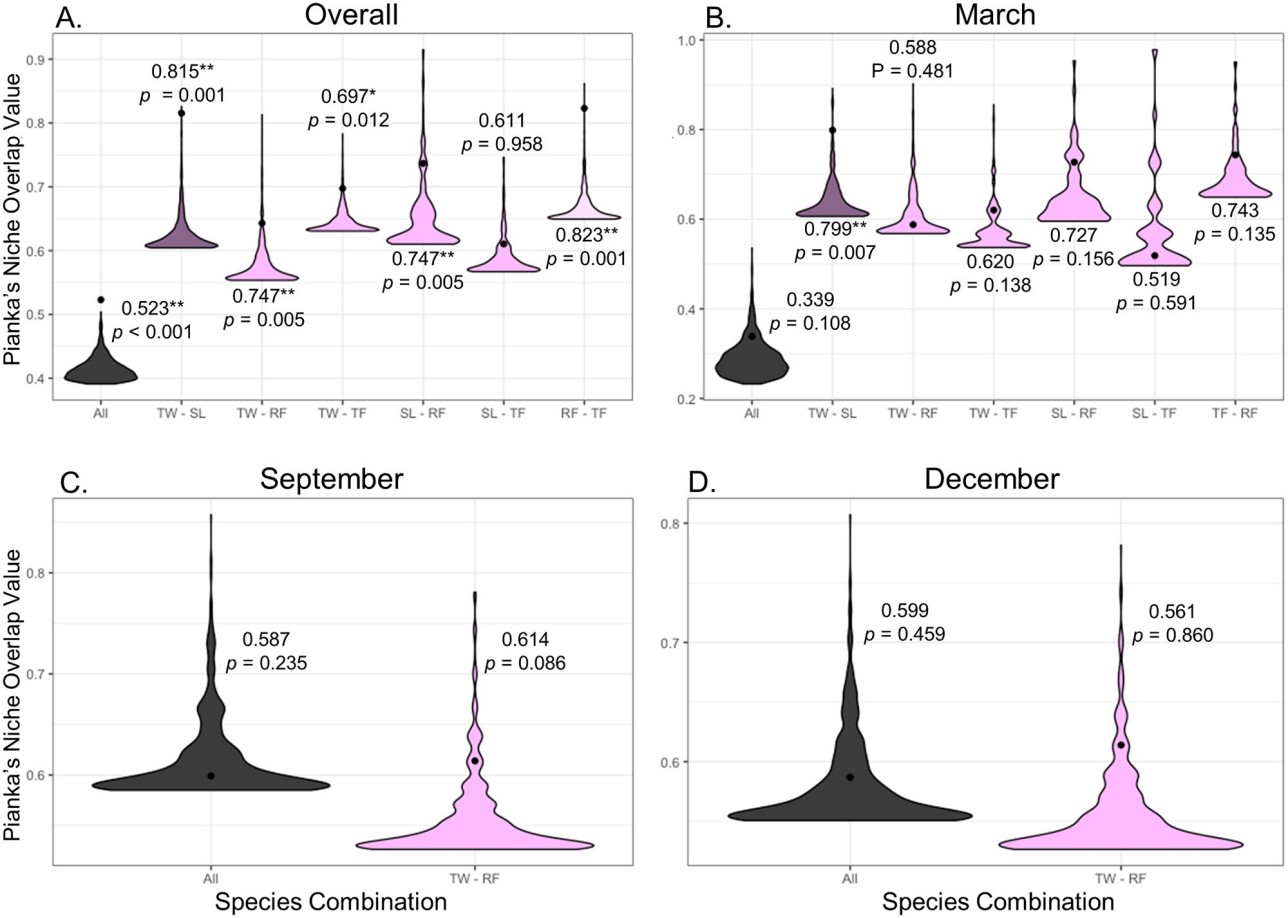
Violin plots representative of dietary niche overlap derived from Pianka’s index values for all host predator samples collected in September 2019, December 2019, March 2020, and July 2020 in the Gouli Nature Reserve, Dulan County, Qinghai Province, China, for all host predators identified (All), as well as pairwise comparisons where sample sizes allowed for statistical significance to be determined (TW–Tibetan wolf; SL–snow leopard; RF–red fox; TF–Tibetan fox). The black dot within the violin area represents the Pianka’s index value and the violin area constitutes the estimated distribution of the index value under the null hypothesis. All predators under consideration are denoted by black coloration, pairwise comparisons between apex predators are denoted by purple, and pairwise comparisons between mesocarnivores are denoted by pink. (A) All data points regardless of collection month. (B) Data from prey determined in scats collected in March. (C) Data from prey determined in scats collected in September. (D) Data from prey determined in scats collected in December.

Beech marten, Pallas’s cat, and Eurasian lynx were removed from Pianka’s index calculations due to low sample sizes. Dietary niche overlap was greater than expected among host predator species regardless of collection month (Pianka’s index = 0.523, *p* < 0.001), and between multiple pairs of host predator species with the exception of snow leopard and Tibetan fox (Fig 5A, S7A Table). Dietary niche overlap was not significantly different than expected based on the null model for March with the exception of Tibetan wolf and snow leopard (Pianka’s index = 0.799, *p* = 0.007) (Fig 5B, S7B Table). Only Tibetan wolf and red fox could be compared for September and December, both of which showed similar dietary overlap, albeit not significantly different than expected based on the null model (Fig 5C and 5D, S7C and S7D Table).

## Discussion

In the Gouli Nature Reserve (GNR), 8 predator species were identified out of 511 scat samples (range: 1–252 scat samples per species) across four seasons (range: 130–188 per season) with a total 26 unique prey items identified (range: 9–17 per season). Species found in diets of 5 or more host predators included blue sheep, Himalayan marmot, and pika. Statistically significant differences were not observed between frequency of occurrence (FOO) and biomass data. The dominant prey by FOO and biomass overall were blue sheep and pika. Dietary diversity metrics were highest among known dietary generalists, the Tibetan wolf and red fox.

Statistically significant differences in seasonal diet were found for the red fox between March and December. Dietary similarity was lowest between the Tibetan wolf and Tibetan fox, highest between the Tibetan fox and red fox, and found to be relatively consistent between any species pair regardless of season, with some exceptions. Dietary niche overlap was greater than expected overall regardless of sample collection month, but not greater than expected overall when examined by host predator species pairs by season with the exception of the Tibetan wolf and snow leopard in March.

### Dietary composition

When considering the dietary composition of all host predator species, small mammals, particularly pika, dominated FOO (Table 1). The FOO of pika ranged from 11.8% for Tibetan wolf to 90% in Pallas’s cat. Wild ungulates were second for FOO but first for biomass, and were almost exclusively comprised of blue sheep. The FOO of blue sheep was highest overall at 59.6% for snow leopard but as low as 0% for Pallas’s cat and beech marten. Blue sheep and pika are ubiquitous and abundant in the GNR and have been noted as important prey for predators in previous work [26]. Pika may be particularly important for sustaining mesopredators and supplementing apex predators because, unlike Himalayan marmot, pika do not hibernate and are available year-round [59]. Many prey species appeared to be taken opportunistically due to low abundance or natural histories and ecological attributes that may make them less accessible (Table 1, S4 Table). For example, voles are small (35 g) and, similar to zokors (*Eospalax* spp.), burrow into the ground [60]. Even ground dwelling bird species (i.e., Galliformes) have the ability to use flight to escape predation. Other prey species dominated in only one host predator, such as woolly hare in Eurasian lynx. Previous studies have found hare FOO values of 22.9% for lynx in southwestern Norway [61] and 91.7% in Turkey [62]. Diet analysis of the Eurasian lynx in mountainous regions of Europe has found high occurrences of ungulates, such as roe deer (*Capreolus capreolus*), indicating that they are capable of hunting similar species, like blue sheep [63]. Evidence of potential intraguild predation was found in the dataset, but was uncommon. Mesopredators in apex predator diet has been observed previously, including wolves predating coyotes, and fishers (*Pekania pennanti*) being killed by coyotes, wolves, and bobcats (*L*. *rufus*) [64]. Determining the true extent of intraguild predation is difficult, as cases could be due to contamination. For example, the presence of domestic dog (*C*. *familiaris*) DNA found in red fox scat was attributed to coprophagia [65].

Herders on the QTP have expressed concern over losing livestock to predators, particularly sheep [66]. Livestock identified in host predators included domestic yak, domestic camel, domestic goat, and domestic sheep. In comparison to domestic goats (28.5 kg) [67] and sheep (36.81 kg) [68], domestic yak are large (304 kg) [69] and often left to roam freely and feed on the grassland with the exception of calves, who are born in early summer and kept near the herder’s home [24]. While safe from most predators, adult yak may still be vulnerable to pack animals, such as Tibetan wolves, who weigh 35 kg [70]. This is similarly echoed in the presence of domestic camel (495 kg) and white-lipped deer (135 kg) [71] in Tibetan wolf diet. Multiple Tibetan wolves and other predators could feed multiple times off one adult carcass over an extended period of time, making larger adult prey a lucrative food resource.

### Dietary diversity

The Tibetan wolf and red fox, known generalist species, had high dietary diversity, while species previously assessed as near-specialists, the snow leopard and Tibetan fox, had relatively low dietary diversity. Evenness, or the number of each prey species consumed, in the Shannon-Wiener index and Simpson’s index may reduce the interpretation of a larger dietary breadth than considering richness alone. For example, in addition to pika, the Tibetan fox consumed 7 other prey species. The snow leopard had a high FOO and biomass of blue sheep with only 4 other prey species noted in this work, but a study involving 191 snow leopard scat samples collected in on the QTP identified 14 different prey species while another involving 165 snow leopard scats collected across multiple regions identified 15 [5, 26].

Low sample sizes may further reduce breadth captured. Molecular dietary work over a larger area overlapping the GNR found prey species in Eurasian lynx scat samples not found in this study [26]. Studies with similarly low sample sizes of Pallas’s cat concluded vastly different metrics surrounding dietary diversity. The ten Pallas’s cat samples in this study exclusively contained pika. Alternatively, a Pallas’s cat diet study in southwestern China involving 14 samples identified 18 different prey items [72]. Differences may be reflective of an abundance of pika or a lack of availability or accessibility of diverse prey in the GNR. Methodological differences between the above-mentioned study and the present study are unlikely given that the same DNA metabarcoding approach was used. The use of DNA metabarcoding versus other dietary analysis methods, such as microhistology, may limit observed dietary diversity. The 12S rRNA genetic marker does not sequence plants or insect species. The consumption of insects, fruits, and seeds during some parts of the year by red foxes may help limit their competition with Tibetan foxes [35]. Seasonally, Pallas’s cats in Mongolia decreased their consumption of pika and increased their consumption of insects in winter, likely because more dead or dormant insects were available [48].

### Seasonal differences in diet

Dietary differences across seasons were not statistically significant overall, indicating that the diet of this predator guild overall does not drastically change over the course of a year. The Tibetan wolf was the only host predator species with sufficient samples to compare dietary changes in FOO across all 4 collection months, which was found to be statistically insignificant. This is in alignment with previous work studying wolf diet in the United States [73], but contrasts work in Nepal [74]. The snow leopard had sufficient samples to compare March and September, but these were found to not be statistically significantly different from another. This coincides with previous work that did not find season to be a predictor of snow leopard diet [74]. Tibetan fox and red fox had sufficient samples sizes to compare diet across 3 collection months. Statistical significance was not found for the Tibetan fox for March, July, and December, but was found for red fox in March, September, and December. Further analyses revealed that differences between the months of March and December were driving this observed difference. March represents samples deposited over the winter months when environmental productivity is low. The red fox may become reliant on more common species, like blue sheep, as other small mammals burrow underground and birds migrate from their breeding grounds. Yak and domestic goat carcasses may become more widely available over the winter months as individuals are slaughtered in November and December or die from harsh conditions, providing more scavenging opportunities [24].

Blue sheep constituted a large proportion of Tibetan wolf diet for FOO, except for September, which was dominated by Himalayan marmot. A similar pattern was found for snow leopard. Blue sheep constituted nearly three-quarters of snow leopard diet FOO in March, but just over one-quarter in September. Marmots on the QTP begin hibernating in mid-October, end their hibernation period in March or early April, and give birth in late spring and early summer [75]. Tibetan wolves and snow leopard may have taken advantage of the influx of marmot during these months. Interestingly, Himalayan marmot was consumed by the Tibetan fox in every month except September, possibly due to increased competition with apex predators. How Tibetan foxes procure marmots while they are presumably hibernating in December and March is unknown. Foxes are known to cache meat at den sites and therefore may have access to previously killed prey items [76, 77]. Himalayan marmots may also reduce or not go into hibernation if temperatures are too warm. Numerous studies have concluded the QTP is warming [29, 78]. One study in Colorado, USA using data from 1976 to 1999 found that yellow-bellied marmots (*M*. *flaviventris*) were appearing above ground 38 days earlier in response to warmer temperatures [31].

Among the four host predator species that consumed livestock, FOO was highest in March for the Tibetan wolf, snow leopard, and red fox and highest in July for the Tibetan fox. March was also the only sampling month to have livestock present in all four host predator species. Predator species may take advantage of easier to procure food resources or become more emboldened to pursue alternative prey when ecological productivity is low. In contrast, FOO was lowest in September for all 4 host predator species. Samples collected in September are representative of a time period of high ecological productivity, whereby predator species may have greater access to native prey species.

### Dietary similarity

Jaccard’s similarity index values are calculated using binary data based on species presence or absence in diet and does not account for the frequency of a species in diet. Overall dietary similarity ranged from 0.353 between the Tibetan wolf and Tibetan fox to 0.615 between the Tibetan fox and red fox (Table 2). The red fox is a generalist while the Tibetan fox is more specialized but high similarity would nonetheless be expected because they are ecologically similar. However, the month of December revealed a much lower similarity value between the two (0.091), likely driven by the red fox’s generalist habits and greater exploitation of less commonly consumed prey. The highest observed similarity value was between the Tibetan wolf and red fox in March. This could be due to a decrease in the availability or accessibility during time periods of lower environmental productivity, and the ability of generalists to consume less preferred prey species. The similarity between the Tibetan wolf and red fox in September, a month reflective of a time with high environmental productivity, was much lower. Dietary similarity between the Tibetan wolf and snow leopard was similar in March and September, suggesting that they exploit similar prey species year-round despite differing environmental conditions.

### Dietary niche overlap

Dietary niche overlap was greater than expected when considering 4 host predator species (Tibetan wolf, snow leopard, Tibetan fox, and red fox) irrespective of season. Statistically significant dietary niche overlap was found for any pair of host predator species irrespective of season with the exception of the snow leopard and Tibetan fox. The dietary niche of mesocarnivores is often nested within that of apex predators, causing a high degree of dietary niche overlap [27]. However, this may not hold true for more specialized species with lower dietary diversity that skews towards a singular species such as the snow leopard with blue sheep and the Tibetan fox with pika. The only month with sufficient sample sizes to compare niche overlap was March. Results showed greater than expected overlap between Tibetan wolf and snow leopard. Previous studies have shown these species to have high overlap, especially in winter [36, 79]. Other studies have that wolves prefer plains-dwelling species while snow leopards prefer cliff-dwelling [74]. FOO data for Tibetan wolf and snow leopard in March reveal that overlap may be driven by closer percentages of blue sheep, pika, and possibly domestic yak compared to other species pairs. Neither September or December showed greater than expected dietary niche overlap for the Tibetan wolf and red fox. The red fox exhibited a high increase in species richness in September (12 different prey species) and December (11 different prey species) relative to March (6 different prey species), and July (5 different prey species). The red fox may have taken greater advantage of a more ecologically productive environment and opportunistically pursued whatever species was available, separating its dietary repertoire from the Tibetan wolf.

Dietary niche partitioning, overall across seasons and in March, was not significantly higher than expected and is reflective of a high degree of overlap. Certain foraging strategies, such as resource sharing via facilitation, could be drawing predator diets close to one another while minimizing competition in the GNR [24, 29]. Resource sharing opportunities occur when mesocarnivores scavenge on a prey item killed and partially consumed by an apex predator [27]. The analysis of synthesized data from 256 studies found that scavenged ungulates comprised as much as 30% of mesocarnivore diets [10]. In this study, occurrences of blue sheep in red fox dietary FOO were lowest in September in comparison to other months. This decrease coincides with a drop in the occurrence of blue sheep in Tibetan wolf and snow leopard diet in September, who instead took advantage of Himalayan marmot. Fewer kills by Tibetan wolf and snow leopard may have decreased scavenging opportunities. This, in combination with a potential increase in the seasonal availability of prey, may have contributed to the red fox’s increase in dietary richness and diversity in September in comparison to other months as it sought out alternative food sources not consumed in other months, such as Avian species and smaller mammals like the zokor (*Eospalax* sp.) and common shrew (*Sorex* sp.).

### Methodological considerations

DNA from scat is challenged by rapid degradation. The number of Undetermined samples was highest in September, perhaps due to increased temperatures and rain. In this study, all remaining scat portions were marked with nail polish. Only 2 recaptures occurred, both in December for scats sampled in September. Scats in the GNR appear to persist less than 3 months, suggesting that studies in similar environments may not violate the 2-month population closure assumption [25]. The genetic marker used in this study is only suitable for vertebrates and may be unable to differentiate closely related species such as domestic dogs and Tibetan wolves, the former of which can also predate wild fauna and livestock [22, 26, 74]. Stray and feral dogs are not common in the GNR and are not expected to have meaningfully contributed to the dataset. Study sites with stray and feral dogs, or other very closely related species, may have to use additional genetic markers [80]. Cannibalism has been recorded in Eurasian lynx in Turkey, but such occurrences where the predator host and the prey are the same species could not be investigated [81]. Biomass analyses should be interpreted with caution. The equation using gray wolf feeding trials may not be representative of all host predator species investigated. Furthermore, true biomass contribution is unknown because prey age and size cannot be determined from scat. Yearly studies are needed to better discern changes in annual climatic variation between seasons and how this may impact dietary resource use. Lastly, the second half of the study period coincided with the beginning of the COVID-19 pandemic. Strong long-standing relationships allowed for samples to be collected by trained community members in March and July of 2020 [82], but research resources were limited and prey count data could not be collected. More detailed questions requiring data on the abundance and availability of prey could not be tested for this study. The influence of prey availability on predator host diet is a logical next step for better understanding this predator guild, particularly in winter when traditional foraging theories are often challenged. The fact that any data were collected is a testament to the importance local community inclusion, and samples are representative of a unique point in time when many other research efforts were unable to continue. Given that the study site has low human-density, it is unlikely that COVID-19 and changes in human behavior impacted ecological processes and predator-prey relationships.

### Conservation and management considerations

Pika was found to be key prey items given its high FOO in the dietary repertoire of host predator species year-round. Ensuring the persistence of this species is likely a key component to sustaining this predator guild. Pika were once considered grassland pests but are now recognized as important environmental engineers and prey for many predators, thus poisoning has largely subsided [83]. Nonetheless, the practice still occurs in isolated areas and may impact predators dependent on them, such as Tibetan fox and Pallas’s cat. Blue sheep were similarly noted to be an important dietary item, particularly for apex predators and specifically snow leopard. Disease outbreaks in the species have previously occurred in high-mountain environments, such as Pakistan and other areas on the QTP [84]. Though abundant, blue sheep live in terrain that is often inaccessible or difficult for humans to traverse and are skittish, making the study and treatment of diseases difficult. Disease monitoring is likely an important tool to ensure the persistence of blue sheep and thus the host predators that heavily exploit them.

Though prey species of seemingly high importance were found in the dataset, the investigation of dietary diversity noted that host predators may possess the resource exploitation plasticity needed to adapt to a reduction or loss of preferred prey. This was found even for commonly categorized near-specialist species, such as the Tibetan fox, in addition to known generalist species. Though the preservation of species such as the pika and blue sheep are imperative, the resiliency of this predator guild may be higher than initially concluded. However, a number of a factors, such as accessibility and availability of less commonly consumed prey, shifts in competition between host predators, and the temporal scale of alterations in prey base, will play large roles in the degree of host predator adaptability.

Livestock made up more in biomass because of the number of yak. The Tibetan wolf exploited livestock year-round, but their presence in predator diet overall was highest in March and July. This may suggest shifts in the wild prey base (lower environmental productivity in winter) or ease of access due to herder practices (calves are born in spring and yak are more free ranging in summer). Despite their size, yak were found in the diets of mesocarnivores and may play an important role in population maintenance. Occurrences were most likely kills of young individuals or from scavenging and may sustain smaller bodied predators through resource-poor time periods. A red fox in central Mongolia was noted as visiting a yak carcass several times, removing meat, and likely caching pieces from the kill site to consume later [85]. Yak carrion is relatively common because Tibetan beliefs do not promote burial of dead livestock [86]. Other livestock species, such as goat and sheep, were not as highly represented in the dataset. This may be due to more stringent protective practices that limit accessibility, such as a human guarding, because these species are smaller in size and more susceptible to predation and environmental elements. Livestock in diet indicates that strategies to reduce conflict such as preventing access to livestock, non-herding vocational opportunities, and burying of dead livestock are needed. Changes should be gradual to allow predators to adapt and for researchers to monitor impacts. Presently, herders should focus on preventing depredation in the months preceding March and July.

## Conclusions

This study offers insight into predator guild ecology at a small spatial scale. High percentages of 3 key prey species, blue sheep, pika, and Himalayan marmot, accompanied by high dietary niche overlap suggests that spatial and daily temporal differences are more responsible for stable coexistence. However, dietary diversity metrics and observed differences between seasons for given species pairs points to a degree of dietary partitioning between apex and mesocarnivores driven by competition for resources and a potentially high dietary plasticity among more specialized species. These observations insinuate that changes in prey availability or accessibility will impact the entire predator guild. Future research in the area should discern micro-spatial and micro-temporal differences to better understand the roles of spatial and daily temporal differences in predator guild function and coexistence.

## Supporting information

S1 Fig Dietary similarity PCoA plots constructed from Jaccard similarity index values for species where sample sizes allowed for data interpretation. (DOCX)

S1 Table Detailed information on the ten transects samples within the study site for molecular dietary analysis of host predator species. (DOCX)

S2 Table The name of each species and its grouping by secondary and tertiary clustering for data analysis and interpretation. (DOCX)

S3 Table The underlying dataset used to reach conclusions regarding the diet of host predator species on the Qinghai-Tibetan Plateau across four sampling months (September 2019, December 2019, March 2020, July 2020) using DNA metabarcoding. (XLSX)

S4 Table The determined mass of an average adult sized individual for each prey item and the source of the metric. (DOCX)

S5 Table Observed as well as lower and upper confidence limits of sampling completeness overall and by month for host predator species with at least one month containing a sufficient number of samples. (DOCX)

S6 Table The percentages of biomass contribution for prey items in the diets of eight predator species across four seasons. (DOCX)

S7 Table Pianka’s index values and level of significance for predator species where sample size was sufficient for comparisons. (DOCX)
