# Supplementary material for: Understanding resource use and dietary niche partitioning in a high-altitude predator guild using seasonal sampling and DNA metabarcoding
Source: PLoS One. 2024 Dec 19;19(12):e0315995. doi: 10.1371/journal.pone.0315995 (PMC11658502; doi:10.1371/journal.pone.0315995)
Supplement: S1 Fig — (DOCX) [file pone.0315995.s001.docx]

Supporting Information S1 Fig. Dietary similarity PCoA plots constructed from Jaccard coefficients for species where sample sizes allowed for data interpretation A) all data points regardless of month; B) for March; C) for September; and D) for December.

Jaccard’s similarity index was calculated based on binary presence or absence of prey species for a particular identified host predator species. This index weighs common and rare prey species in samples equally and is calculated via the following formula:

$$J(A,B)=\frac{| A\cap B |}{| A\cup B |}$$

Where $A\cap B$ represents the prey species that both predator species A and predator species B have in common and $A\cup B$ represents all prey species in the dietary repertoire of predator species A and predator species B. The index ranges from 0 to 1, with 1 indicating complete similarity and 0 indicating complete dissimilarity. Points spatially closer to one another in their respective PCoA plot indicate greater similarity.
