# Supplementary material for: Understanding resource use and dietary niche partitioning in a high-altitude predator guild using seasonal sampling and DNA metabarcoding
Source: PLoS One. 2024 Dec 19;19(12):e0315995. doi: 10.1371/journal.pone.0315995 (PMC11658502; doi:10.1371/journal.pone.0315995)
Supplement: S1 Table — (DOCX) [file pone.0315995.s002.docx]

Supporting Information S1 Table. Detailed information on the ten transects samples within the study site for molecular dietary analysis of host predator species.

| **Transect Number** | **Sampling Area** | **GPS Location Start** | **GPS Location End** | **Elevation Range (m)** | **Distance (km)** | **Dominant Topographic Features** | **Landform Ruggedness** | **Grazing Status** |
| --- | --- | --- | --- | --- | --- | --- | --- | --- |
| 1 | Ren Long Cun of Gouli | 35.64816°E, 98.47176°N | 35.64738°E, 98.49340°N | 3,938 – 4,429 | 2 | Ridgeline; Hillside | Very Broken/Steep | Seasonal - Livestock kept in winter |
| 2 | Ren Long Cun of Gouli | 35.64819°E, 98.471706°N | 35.6467°E, 98.47106°N | 3,921 – 4,200 | 2.3 | Hillside | Very Broken/Steep | Seasonal - Livestock kept in winter |
| 3 | Ren Long Cun of Gouli | 35.65337°E, 98.49767°N | 35.65968°E, 98.50040°N | 4,311 – 4,449 | 2 | Ridgeline; Hillside | Moderately Steep | Seasonal - Livestock kept in winter |
| 4 | Delong Gou | 35.51239°E, 98.34261°N | 35.53093°E, 98.43856°N | 4,458 – 4,679 | 3.5 | Ridgeline | Moderately Steep | Seasonal - Livestock kept in summer |
| 5 | Delong Gou | 35.5315°E, 98.3306°N | 35.5245°E, 98.3334°N | 4,531 – 4,700 | 2.2 | Hillside | Moderately Steep | Seasonal - Livestock kept in summer |
| 6 | Delong Gou (Xia La Wen) | 35.52952°E, 98.843827°N | 35.5300°E, 98.4381°N | 4,048 – 4,172 | 1.7 | Ridgeline; River/Drainage Bottom | Moderately Steep | Seasonal - Livestock kept in summer |
| 7 | Duo Jiao Hu | 35.63624°E, 98.51250°N | 35.63624°E, 98.51250°N (same as start - transect followed in oblong loop) | 4,040 - 4,189 | 2.7 | River/Drainage Bottom | Flat | Seasonal - Livestock kept in winter |
| 8 | Duo Jiao Hu | 35.64507°E, 98.52818°N | 35.64507°E, 98.52818°N (same as start - transect followed in oblong loop) | 4,152 - 4,259 | 1.6 | Ridgeline; Hillside; River/Drainage Bottom | Moderately Steep | Seasonal - Livestock kept in winter |
| 9 | Ren Long Cun of Gouli | 35.66530°E, 98.46046°N | 35.674134°E, 98.459936°N | 4,122 - 4,275 | 1.4 | Hillside; River/Drainage Bottom | Rolling | Seasonal - Livestock kept in winter |
| 10 | Re Long Guo | 35.58954°E, 98.40818°N | 35.58211°E, 98.40253°N | 4,016 - 4,174 | 3.1 | Ridgeline; Hillside; River/Drainage Bottom | Very Broken/Steep | Year-Round - Livestock kept in winter and summer |
