# Supplementary material for: Understanding resource use and dietary niche partitioning in a high-altitude predator guild using seasonal sampling and DNA metabarcoding
Source: PLoS One. 2024 Dec 19;19(12):e0315995. doi: 10.1371/journal.pone.0315995 (PMC11658502; doi:10.1371/journal.pone.0315995)
Supplement: S2 Table — (DOCX) [file pone.0315995.s003.docx]

Supporting Information S2 Table. The common name and scientific name of each species and its grouping by secondary and tertiary clustering for data analysis and interpretation.

| **Primary Tier Prey Name** | **Secondary Tier Prey Name** | **Tertiary Tier Prey Name** |
| --- | --- | --- |
| Domestic Yak (*Bos grunniens*) | Domestic Yak | Livestock |
| Domestic Camel (*Camelus bactrianus*) | Domestic Camel |  |
| Domestic Goat (*Capra aegagrus hircus*) | Domestic Goat |  |
| Domestic Sheep (*Ovis aries*) | Domestic Sheep |  |
| Blue Sheep (*Pseudois nayaur*) | Blue Sheep | Wild Ungulates |
| White-lipped Deer (*Cervus albirostris*) | White-lipped Deer |  |
| Tibetan Fox (*Vulpes ferrilata*) | Tibetan Fox | Carnivores |
| Red Fox (*Vulpes vulpes*) | Red Fox |  |
| Himalayan Marmot (*Marmota himalayana*) | Himalayan Marmot | Small Mammals |
| Woolly Hare (*Lepus oiostolus*) | Woolly Hare |  |
| Zokor (*Eospalax* sp.) | Zokor |  |
| Mountain Weasel (*Mustela altaica*) | Mountain Weasel |  |
| Pika (*Ochotona curzoniae*) | Pika |  |
| Long-tailed Dwarf Hamster (*Cricetulus longicaudatus*) | Long-tailed Dwarf Hamster |  |
| Narrow Headed Vole (*Microtus gregalis*) | Vole species |  |
| Grey Red-Backed Vole (*Myodes rufocanus*) |  |  |
| Chinese Scrub Vole (*Neodon irene*) |  |  |
| Sikkim Vole (*Neodon sikimensis*) |  |  |
| Common Shrew (*Sorex* sp.) | Common Shrew |  |
| Upland Buzzard (*Buteo hemilasius*) | Birds of Prey | Birds |
| Falcon (*Falco* sp.) |  |  |
| Common Raven (*Corvus corax*) | Perching Birds |  |
| Brown Accentor (*Prunella fulvescens*) |  |  |
| Pine Bunting (*Emberiza leucocephalos*) |  |  |
| Snowcock (*Tetraogallus himalayensis*) | Ground Feeding Birds |  |
| Chukar Partridge (*Alectoris chukar*) |  |  |
| Undetermined | Undetermined | Undetermined |
