# Supplementary material for: Understanding resource use and dietary niche partitioning in a high-altitude predator guild using seasonal sampling and DNA metabarcoding
Source: PLoS One. 2024 Dec 19;19(12):e0315995. doi: 10.1371/journal.pone.0315995 (PMC11658502; doi:10.1371/journal.pone.0315995)
Supplement: S4 Table — (DOCX) [file pone.0315995.s005.docx]

Supporting Information S4 Table. The determined mass of an average adult sized individual for each prey item and the source of the metric.

| **Prey Species** | **Mean Average Mass (kg)** | **Source** |
| --- | --- | --- |
| Domestic Yak | 304 | Editing Committee [Qinghai] 1983 |
| Domestic Camel | 495 | Animal Diversity Web |
| Domestic Goat (cashmere) | 28.5 | Bai et al. 2006 |
| Domestic Sheep (Tibetan) | 36.81 | Xu et al. 2017 |
| Blue Sheep | 57.25 | Britannica |
| White-lipped Deer | 135 | Animal Diversity Web |
| Tibetan Fox | 4.75 | Harris et al. 2008 |
| Red Fox | 8.1 | Walkers Mammals of the World |
| Himalayan Marmot | 6.6 | Nikol'skii and Ulak 2006 |
| Woolly Hare | 2.3 | Animal Diversity Web |
| Zokor | 0.38 | Smith et al. 2010 |
| Mountain Weasel | 0.23 | King and Powell 2007 |
| Pika | 0.16 | Britannica |
| Long-tailed Dwarf Hamster | 0.032 | Smith et al. 2010 (pg. 243) |
| Narrow Headed Vole | 0.035 | Sheftel and Hentonen 2016 |
| Grey Red-Backed Vole | 0.035 | Sheftel and Hentonen 2016 |
| Chinese Scrub Vole | 0.035 | Sheftel and Hentonen 2016 |
| Sikkim Vole | 0.035 | Sheftel and Hentonen 2016 |
| Common Shrew | 0.008 | Hutterer and Kryštufek 2016 |
| Upland Buzzard | 1.31 | Cui et al. 2008 |
| Falcon | 1 | Shobrak 2015 |
| Common Raven | 1.2 | BirdLife International 2017 |
| Brown Accentor | 0.03 | McClure 1991 |
| Pine Bunting | 0.03 | BirdLife International 2017 |
| Himalayan Snowcock | 3.1 | Chardine and Dunning 1994 |
| Chukar Partridge | 0.61 | Animal Diversity Web |
