# Supplementary material for: Understanding resource use and dietary niche partitioning in a high-altitude predator guild using seasonal sampling and DNA metabarcoding
Source: PLoS One. 2024 Dec 19;19(12):e0315995. doi: 10.1371/journal.pone.0315995 (PMC11658502; doi:10.1371/journal.pone.0315995)
Supplement: S5 Table — (DOCX) [file pone.0315995.s006.docx]

Supporting Information S5 Table. Observed as well as lower and upper confidence limits of sampling completeness overall and by month for host predator species with at least one month containing sufficient sample numbers. - indicates that sample sizes were not sufficient to determine sampling completeness.

|  | Overall | March | July | September | December |
| --- | --- | --- | --- | --- | --- |
| Tibetan  Wolf | 0.962 | 0.953 | 0.971 | 0.759 | 0.919 |
|  | (0.947 - 0.977) | (0.916 - 0.989) | (0.946 - 0.995) | (0.653 - 0.864) | (0.843 - 0.996) |
| Snow  Leopard | 0.935 | 1.000 | - | 0.782 | 0.733 |
|  | (0.879 - 0.992) | (0.938 - 1.000) |  | (0.532 - 1.000) | (0.280 - 1.000) |
| Eurasian  Lynx | 0.817 | 0.333 | 0.750 | - | - |
|  | (0.438 - 1.000) | (0.000 - 0.750) | (0.559 - 0.941) |  |  |
| Tibetan  Fox | 0.940 | 0.831 | 0.891 | - | 0.896 |
|  | (0.889 - 0.992) | (0.704 - 0.957) | (0.785 - 0.996) |  | (0.844 - 0.949) |
| Red  Fox | 0.922 | 0.915 | 0.706 | 0.904 | 0.935 |
|  | (0.891 - 0.953) | (0.828 - 1.000) | (0.517 - 0.895) | (0.847 - 0.962) | (0.890 - 0.979) |
| Beech  Marten | 0.608 | - | - | 0.608 | - |
|  | (0.356 - 0.860) |  |  | (0.338 - 0.877) |  |
