# Supplementary material for: Understanding resource use and dietary niche partitioning in a high-altitude predator guild using seasonal sampling and DNA metabarcoding
Source: PLoS One. 2024 Dec 19;19(12):e0315995. doi: 10.1371/journal.pone.0315995 (PMC11658502; doi:10.1371/journal.pone.0315995)
Supplement: S6 Table — (DOCX) [file pone.0315995.s007.docx]

Supporting Information S6 Table. The sample sizes and percentages (%) of biomass for prey items in the diets of eight predators across four seasons. (O = Overall, M = March, J = July, S = September, D = December). Scientific names are available in S2 Table. Note that Tibetan brown bear and beech marten were excluded due to low samples sizes and available dietary data for only one season.
