# Supplementary material for: Understanding resource use and dietary niche partitioning in a high-altitude predator guild using seasonal sampling and DNA metabarcoding
Source: PLoS One. 2024 Dec 19;19(12):e0315995. doi: 10.1371/journal.pone.0315995 (PMC11658502; doi:10.1371/journal.pone.0315995)
Supplement: S7 Table — (DOCX) [file pone.0315995.s008.docx]

Supporting Information S7 Table. Pianka’s index values and level of significance for predator species where comparisons were permitted by sufficient sample sizes for A) all data points regardless of month; B) samples collected in March; C) samples collected in September; and D) samples collected in December.

A.

|  | All Carnivores Overall | Tibetan Wolf Overall | Snow Leopard Overall | Tibetan  Fox Overall | Red Fox Overall |
| --- | --- | --- | --- | --- | --- |
| All Carnivores Overall | 0.523** |  |  |  |  |
|  | *p* < 0.001 |  |  |  |  |
| Tibetan Wolf Overall |  | - |  |  |  |
|  |  |  |  |  |  |
| Snow Leopard Overall |  | 0.815** | - |  |  |
|  |  | *p* < 0.001 |  |  |  |
| Tibetan Fox Overall |  | 0.697* | 0.611 | - |  |
|  |  | *p* = 0.012 | *p* = 0.958 |  |  |
| Red Fox Overall |  | 0.643 | 0.747** | 0.823** | - |
|  |  | *p* = 0.389 | *p* = 0.005 | *p* < 0.001 |  |

B.

|  | All Carnivores March | Tibetan Wolf March | Snow Leopard March | Tibetan  Fox March | Red Fox March |
| --- | --- | --- | --- | --- | --- |
| All Carnivores March | 0.339 |  |  |  |  |
|  | *p* = 0.108 |  |  |  |  |
| Tibetan Wolf March |  | - |  |  |  |
|  |  |  |  |  |  |
| Snow Leopard March |  | 0.799** | - |  |  |
|  |  | *p* = 0.007 |  |  |  |
| Tibetan Fox March |  | 0.620 | 0.519 | - |  |
|  |  | *p* = 0.138 | *p* = 0.591 |  |  |
| Red Fox  March |  | 0.588 | 0.727 | 0.743 | - |
|  |  | *p* = 0.481 | *p* = 0.156 | *p* = 0.135 |  |

C.

|  | All Carnivores  September | Tibetan Wolf September | Red Fox  September |
| --- | --- | --- | --- |
| All Carnivores September | 0.587 |  |  |
|  | *p* = 0.235 |  |  |
| Tibetan Wolf September |  | - |  |
|  |  |  |  |
| Red Fox September |  | 0.614 | - |
|  |  | *p* = 0.086 |  |

D.

|  | All Carnivores December | Tibetan Wolf December | Red Fox December |
| --- | --- | --- | --- |
| All Carnivores December | 0.599 |  |  |
|  | *p* = 0.459 |  |  |
| Tibetan Wolf December |  | - |  |
|  |  |  |  |
| Red Fox December |  | 0.561 | - |
